# The Role of the Unbinding Cycle on the Coordination Abilities of the Bi-Cyclopeptides toward Cu(II) Ions

**DOI:** 10.3390/molecules29102197

**Published:** 2024-05-08

**Authors:** Alicja Lisowska, Paulina Świątek, Filip Dębicki, Agnieszka Lewińska, Aleksandra Marciniak, Lorenzo Pacini, Anna Maria Papini, Justyna Brasuń

**Affiliations:** 1 Biomolecule Student Science Club, Faculty of Pharmacy, Wroclaw Medical University, 50-556 Wroclaw, Poland; alicja.lisowska@student.umw.edu.pl; 2 Graduate of Faculty of Pharmacy, Wroclaw Medical University, 50-556 Wroclaw, Poland; paulina.swiat97@gmail.com; 3 Faculty of Medicine, Medical University of Lodz, 90-647 Lodz, Poland; filip.debicki97@gmail.com; 4 Faculty of Chemistry, Wroclaw University, 50-137 Wroclaw, Poland; agniezska.lewinska@uwr.edu.pl; 5 Department of the Basic Chemical Sciences, Wroclaw Medical University, 50-556 Wroclaw, Poland; aleksandra.marciniak@umw.edu.pl; 6 Interdepartmental Research Unit of Peptide and Protein Chemistry and Biology, University of Florence, Sesto Fiorentino, 50019 Florence, Italy; l.pacini@unifi.it; 7 Department of Chemistry “Ugo Schiff”, University of Florence, Sesto Fiorentino, 50019 Florence, Italy

**Keywords:** bi-cyclopeptide, histidine, metal complex, copper ions, stability constant, spectroscopic studies

## Abstract

Bicyclic peptides have attracted the interest of pharmaceutical companies because of their remarkable properties, putting them on a new path in medicine. Their conformational rigidity improves proteolytic stability and leads to rapid penetration into tissues via any possible route of administration. Moreover, elimination of renal metabolism is of great importance, for example, for people with a history of liver diseases. In addition, each ring can function independently, making bicyclic peptides extremely versatile molecules for further optimization. In this paper, we compared the potentiometric and spectroscopic properties studied by UV–vis, MCD, and EPR of four synthetic analogues of the bi-cyclic peptide c(PKKHP-c(CFWKTC)-PKKH) (BCL). In particular, we correlated the structural and spectral properties of complexes with coordinating abilities toward Cu(II) ions of MCL1 (Ac-PKKHPc(CFWKTC)PKKH-NH_2_) that contains the unbinding cycle and N- and C-terminal linear parts with two histidine residues, one per part; two monocyclic ligands containing one histidine residue, both in the N-terminal position, i.e., MCL2 (Ac-PKKHPc(CFWKTC)PKKS-NH_2_) and in the C-terminal position, i.e., MCL3 (Ac-PKKSPc(CFWKTC)PKKH-NH_2_), respectively; and the linear structure LNL (Ac-PKKHPSFWKTSPKKH-NH_2_). Potentiometric results have shown that the bicyclic structure promotes the involvement of the side chain imidazole donors in Cu(II) binding. On the other hand, the results obtained for the mono-cyclic analogues lead to the conclusion that the coordination of the histidine moiety as an anchoring group is promoted by its location in the peptide sequence further from the nonbinding cycle, strongly influencing the involvement of the amide donors in Cu(II) coordination.

## 1. Introduction

Cyclic peptides include in their structure or are themselves rings of polypeptide chains, which can be synthesized by selective side-chain-to-side-chain or head-to-tail cyclization strategies, respectively. Homodetic cyclopeptides are based on lactam analogues, compared to heterodetic ones based, for example, on the classical side-chain to side-chain disulfide bridges but also on other proteolytically stable non-native bonds such as triazolyl bridges [1].

The cyclization affects the stiffening of the structure [2] and allows the mimicry of the secondary structure of some protein fragments [3]. Moreover, the cyclic structure promotes the anchoring of metal ions by amino acid side chains [4]. Studies have proven that cyclic peptides show specificity in forming bonds with certain metal ions. Experiments with native cyclopeptides, such as oxytocin and vasopressin, have proven that despite their lack of coupling to metal ions in nature, they show high ability and efficiency to form bonds with particular metal ions [5,6,7,8,9,10,11]. 

There are many cyclic peptides displaying antibacterial, anticancer, immunosuppressive, or toxic properties [12,13], and they are increasingly used in medicine, showing better biological activity than linear peptides [14]. Moreover, some natural bicyclic peptides that are known to be produced by different organisms, such as romidepsin, actinomycin D, moroidin, and celogentin C, are known for different activities, such as inhibition of tubulin polymerization, prevention of actin filament polarization, and inhibition of RNA polymerase II, among others [15,16,17]. Interestingly, the concept of Bicycles^®^ [18] attracted more and more pharmaceutical companies as synthetic bicyclic peptides were rotationally limited by symmetrical small-molecule scaffolds that constrain the molecule into rigid two-loop structures. Their versatile peptide scaffold allows iterative and fast optimization processes to increase affinity and selectivity for difficult-to-drug targets. Bicycles are made stable through optimization, and their low molecular weight delivers favorable pharmacokinetics, enabling deep tissue penetration, high solubility, and flexibility in the areas of manufacturing, route of administration, and formulation. The routes of administration of drugs containing bicyclic peptides feature a wide range of choices. Their additional advantage is their rapid penetration into tissues. Furthermore, elimination occurs through renal metabolism, which can be of great importance in the use of these compounds in patients with a history of liver disease. The advantage of bicycle peptides is their size and increased conformational rigidity, which improves proteolytic stability in the blood. In addition, each ring can function independently of the others. Moreover, studies point to their use as platforms due to their biocompatibility, similarity, and chemical diversity with previously known native proteins [19,20]. 

The presence of a second cycle results in an even greater increase in biological stability and ability to bind to the receptor, as each ring in the bicyclic structure can function independently, which makes these peptides bifunctional [21]. Thus, bicyclic peptides offer high expectations for the development of new drug candidates carriersdue to their structural similarity with proteins [22]. 

In this paper, we focused on dissecting the binding abilities of bicyclic peptides toward Cu(II) ions. This microelement is essential for living systems; e.g., it is involved in several enzymatic processes, but it is also relevant for antioxidant properties. Cu(II) transport, storage, and biological activity are related to its interaction with peptides and/or proteins.

Previous studies on the coordination properties of a bicyclic peptide with one non-binding cycle and a second cycle with two histidine residues as anchoring groups have shown that the bicyclic structure strongly influences the efficacy of the Cu(II) coordination [23], possibly related to the H-bond formation [24].

The investigations of the binding abilities of other bicyclopeptides with the c(PKKHP-c(CFWKTC)-PKKH) (BCL) sequence have shown that, above pH 7.4, the peptide forms copper complexes with the untypical transition at 483 nm in the UV–vis spectra [25]. Owing to this fact, we propose herein the results of the studies of four different monocyclic analogues of the original bicyclopeptide BCL. The first, MCL1 (sequence: Ac-PKKHPc(CFWKTC)PKKH-NH_2_, Bachem AG, Bubendorf, Switzerland), is composed of the unbinding cycle and two linear parts, N- and C-terminal, with two histidine residues, one per part. The next two monocyclic ligands contain one histidine residue in the N-terminal (MCL2: Ac-PKKHPc(CFWKTC)PKKS-NH_2_) or C-terminal positions (MCL3: Ac-PKKSPc(CFWKTC)PKKH-NH_2_), respectively, and the last ligand has a linear structure (LNL: Ac-PKKHPSFWKTSPKKH-NH_2_).

**Scheme 1:**
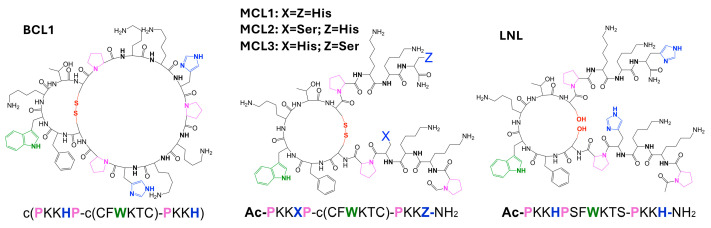
Structures of the synthetic peptide ligands BCL, MCL1, MCL2, MCL3, and LNL. The potential binding sites relevant for Cu(II) are: Pro residues colored in pink (allowing to receive the independent binding sites for the metal ion); the imidazole ring of His in blue (anchoring group for the metal ion); the indole ring of Trp in green (investigated by MCD); the S-S bond, forming the cyclic structure in BCL; MCL1, MCL2, MCL3 in red; and the hydroxyl side chains of Ser, replacing Cys to obtain the linear analog LNL in red.

In Scheme 1, the structures of the synthetic peptide ligands BCL, MCL1, MCL2, MCL3, and LNL are reported, along with the potential binding sites relevant for Cu(II) complex formation.

## 2. Results and Discussion

The analyses of the acid-base properties of MCL1 allowed for the determination of all protonation constants related to the five side chains of Lys residues and two of the imidazole rings of both His residues (Table 1). The values of all protonation constants are comparable to those previously obtained for BCL [25].

**Figure 1:**
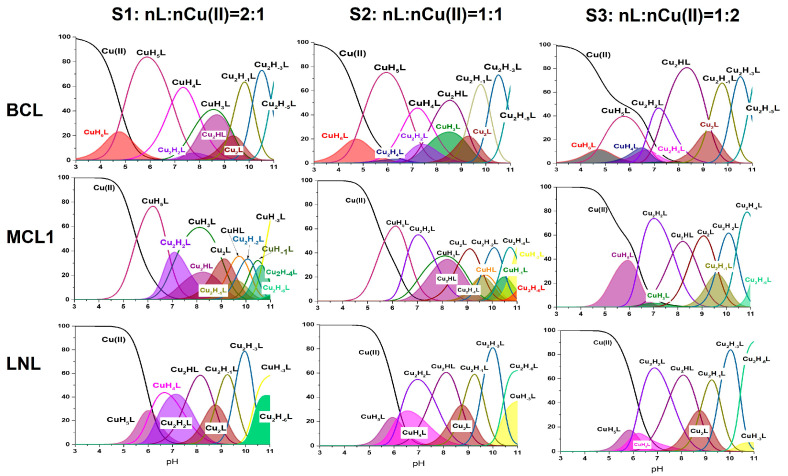
The coordination profile for BCL, MCL1, and LNL.

The MCL1 and LNL peptides have in their structure the same type and number of deprotonating groups as BCL. Due to this fact, the stoichiometry of the formed complexes can be compared. Both peptides have two separated His residues, and based on the fact that imidazole nitrogen is the anchoring group for Cu(II) ions, three systems with nL:nCu(II) = 2:1 (S1), nL:nCu(II) = 1:1 (S2), and nL:nCu(II) = 1:2 (S3) of MCL1 and LNL were investigated with potentiometry. Both MCL1 and LNL form mono- and di-nuclear complexes as BCL (Table 1, Figure 1). 

MCL1, as well as LNL, form the CuH_5_L species as the first complex. The appearance of this complex in both systems is related to the dissociation of two protons from the ligand molecules. In the case of BCL, CuH_5_L is characterized by the {2 N_Im_} binding mode [25]. The value of the calculated stability constants depends on the protonation state of the ligand. It is therefore more advantageous to compare the values of the constants independently of the protonation of the ligand, calculated as logβ* = *logβ_CuHnL_* − *logβ_HmL_*_._ The values of the corrected stability constants, logβ*_MCL1_ = 4.52 and logβ*_LNL_ = 4.08, are significantly lower than logβ*_BCL_ = 5.68, which supports the involvement of only one imidazole donor in copper binding [26]. In contrast to BCL, which forms the final mononuclear species CuH_3_L with the {N_Im_, 2N_amide_} coordination manner, MCL1 and L form the CuH_−3_L species with the {N_Im_, 3N_amide_} binding mode.

MCL1, in both linear parts, has His residues, and due to this fact, it is difficult to define which linear part (N- or C-terminal) favors copper coordination. Because of this, its binding abilities were compared to the analogues having one His residue in the structure (MCL2 in the N-terminal part and MCL3 in the C-terminal part). MCL2 and MCL3 form the CuH_4_L complex as the first. Its formation, as it was found, is related to the dissociation of two protons. The value of the corrected stability constant (logβ*_MCL2_ = 5.27 and logβ*_MCL3_ = 6.16) is significantly higher than the stability of MCL1. This result strongly supports different binding modes, i.e., one imidazole and one amide moiety. 

Both MCL2 and MCL3 form the final species with the {N_im_, 3N_amide_} binding mode that was observed in the MCL1 system. The stability of this species formed by MCL1 is almost the same as the stability of MCL3 (Table 2), which strongly suggests that the linear C-terminal part of the MCL1 sequence is favored. 

The di-nuclear complexes existing in the studied system are the second group. Similarly to the BCL systems, the formation of these complexes is promoted with an increase in the Cu(II) concentration concerning the ligand concentration.

**Figure 2:**
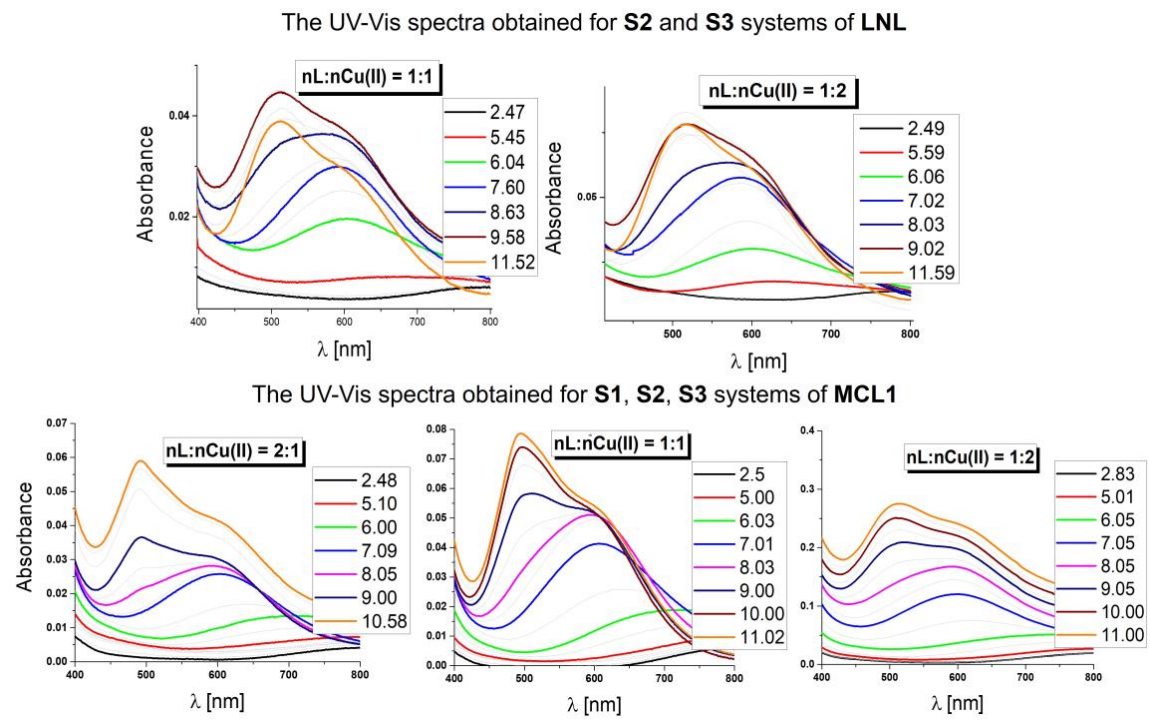
The pH-dependent absorption and UV–vis spectra of the LNL peptide: S2 and S3 systems and of the MCL1 peptide: S1, S2, and S3 systems.

As it was mentioned above, in the UV-vis spectra of all BCL systems [25], there is an untypical transition at 483 nm. Figure 2 shows the UV-vis spectra obtained for the MCL1-Cu(II) in the systems with the 2:1 (S1), 1:1 (S2), and 1:2 (S3) molar ratios and the 1:1 (S2) and 1:2 (S3) molar ratios systems of the LNL.

The absorption spectra obtained for both systems of the linear peptide LNL are typical of copper complexes of the peptide and are in good agreement with the potentiometric results. Analyzing the absorption spectra obtained for the MCL1 peptide, we observed that the spectra of system S3 are similar to those obtained for the LNL. The difference can be observed for the two first systems (S1 and S2) with transitions below 500 nm (what was observed for the BCL [25]).

The transition located below 500 nm appears to be above pH 8. In these conditions, in both systems, two complexes dominate, i.e., the mononuclear CuH_3_L and the di-nuclear Cu_2_HL. The concentration of CuH_3_L decreases with the change of the molar ratio MCL1:Cu(II) = 2:1 > 1:1 (in the system with the molar ratio MCL1:Cu(II) = 1:2, its concentration = 0), and it can be assumed that the presence of the transition below 500 nm is determined by the mononuclear species. 

**Figure 3:**
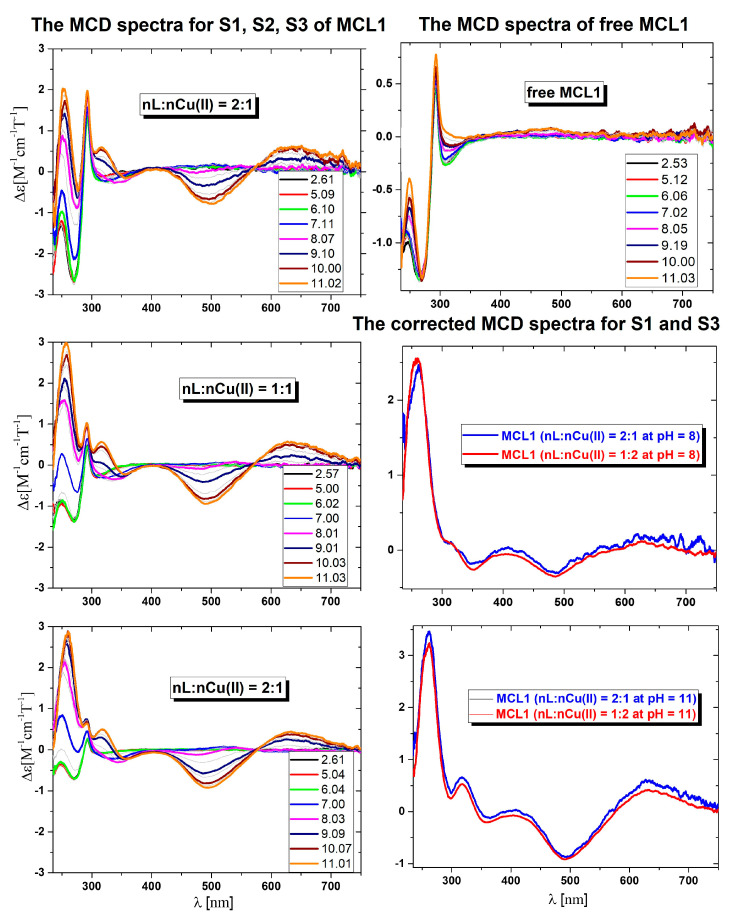
The pH-dependent MCD spectra of the MCL1 peptide in all systems, of the free MCL1 peptide, and the corrected MCD spectra obtained at pH 8 and 11.

In the unbinding cycle of BCL as well as the MCL1 peptide, the Trp moiety is present. Based on the fact that Trp gives the characteristic transition in the MCD spectra, all MCL1-Cu(II) systems were investigated with MCD (Figure 3).

In all systems with metal ions, there are two transitions, i.e., one positive at 650 nm and one negative at 500 nm, which are related to the d-d transitions. Below 400 nm are present the transitions characteristic for Trp, CT_Nim → Cu(II)_, and CT_Nim → Cu(II)_. The pH-dependent changes in the MCD spectra correlate well with the potentiometric results. 

The detailed analysis of the Trp region of the Cu(II) complexes concerning the free ligand may give information on the possible interaction of Cu(II) with the Trp side chain. Based on the potentiometric results, the MCD spectra were analyzed at pH 8 and 11 for S1 and S3 molar ratios.

At pH 8, in the first system (S1), two main complexes exist, i.e., the dominant CuH_3_L and Cu_2_HL, while in the system with nL:nCu(II) = 1:2 (S3), the dominant is Cu_2_HL. In the CuH_3_L species, the metal ion is coordinated by three N-donors: one imidazole and two amides. The formation of the Cu_2_HL complexes is related to the loss of six protons from the ligand molecule. Based on the fact that in these conditions all Lys residues are protonated, there are two main possibilities for binding of both Cu(II) ions: {1N_Im_, 1N_amide_}/{1N_Im_, 3N_amide_} or {1N_Im_, 2N_amide_}/{1N_Im_, 2N_amide_}. At pH 8.5, the corrected MCD spectra (complex spectrum–ligand spectrum) of both systems are almost the same. This result strongly supports the same binding mode of copper ions in the mono- as well as in the di-nuclear complexes (Figure 3). A similar situation is observed at pH 11, where the final complexes are formed. In the first system, the CuH_−3_L species dominates, while in the second, the Cu_2_H_−5_L complex dominates. The corrected spectra are the same, thus confirming the same {1N_Im_ and 3N_amide_} donors in the coordination sphere of the Cu(II) ions.

**Figure 4:**
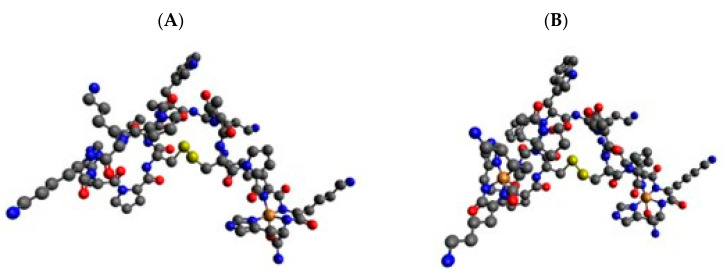
Structures of the final complexes: the mononuclear CuH_−3_L (panel **A**) and di-nuclear Cu_2_H_−6_L (panel **B**) of MCL1 (L). Cu(II) ions are marked in orange, the N-atoms are marked in blue, the O-atoms are marked in red, and the S-atoms are marked in yellow. The structures were obtained with the Avogadro program [27,28].

The proposed structures of the final mono- and di-nuclear complexes are presented in Figure 4 (panels A and B). 

Moreover, the region characteristic for the Trp residue is not affected, which strongly supports that the bound Cu(II) ions do not affect the Trp residue, and the presence of this additional transition below 500 nm is not related to an interaction of the side chain of Trp with metal ions. 

The next step of the presented studies was the analysis of the EPR spectra for the BCL-Cu(II) and MCL1-Cu(II) systems. 

**Figure 5:**
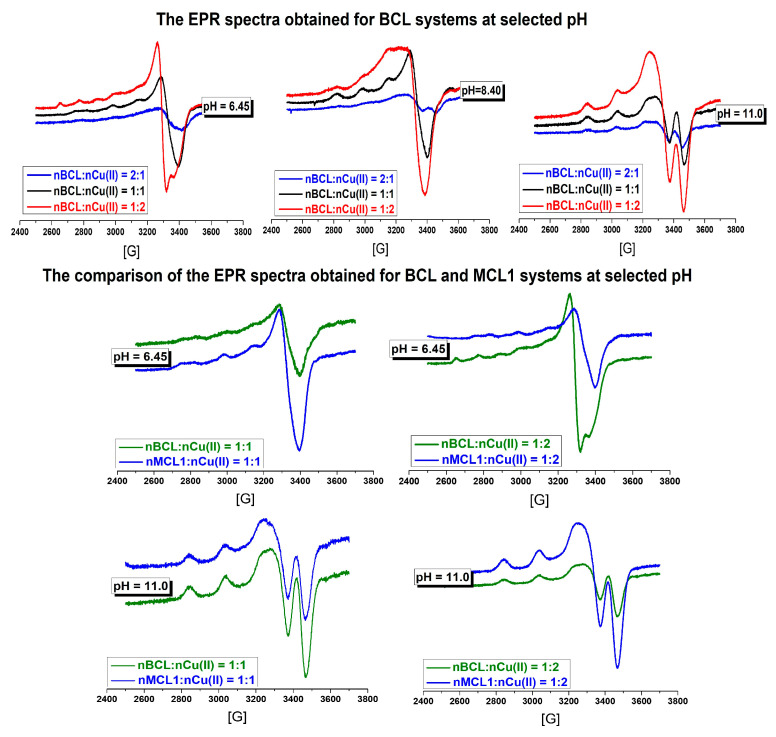
The pH-dependent EPR spectra of the BCL peptide in all systems obtained at selected pH and comparison of the EPR spectra of the BCL and MCL1 peptides in the S1 and S3 systems obtained at pH 6.45 and 11.

The investigated BCL-Cu(II) systems were systems with all three molar ratios at pH 6.45, 8.4, and 11. The comparison of the EPR spectra obtained for all BCL systems at pH = 6.45 is shown in Figure 5. At this pH in all systems, the CuH_5_L species is dominant, but other complexes are also present. Due to this fact, it is difficult to obtain the EPR parameters for this species. However, in the system with a 1:2 molar ratio, EPR parameters characteristic of the Cu(II)_aq_ species can be identified, in good agreement with the potentiometric results. The EPR spectra from pH 8.4 (Figure 5) of all BCL systems are very similar with A_II_ = 169 G and g_II_ = 2.234 parameters and support the presence of three N-donors in the coordination sphere of the Cu(II) ion. The EPR parameters A_II_ = 192 G and g_II_ = 2.187 found at pH 11 confirm the existence of the 4N-type of the complex.

In relation to our previous results [25], the untypical transition below 500 nm is present in all systems of MCL1. Due to this fact, two MCL1-Cu(II) systems, i.e., nL:nCu(II) = 1:1 and nL:nCu(II) = 1:2, were investigated and compared to BCL in the same conditions. The comparison of the spectra of the investigated systems around pH = 6.45 as well as pH = 11 is very similar and supports the same number of N−donors and a similar environment of the metal ion. These results suggest that the presence of the transition below 500 nm is not related to the interaction of the additional group with the metal ion.

## 3. Materials and Methods

### 3.1. Synthesis of the Peptides LNL, MCL2, and MCL3

Materials for Peptide Synthesis

Fmoc-L-Pro-OH; Fmoc-L-Lys(Boc)-OH; Fmoc-L-Ser(tBu)-OH; Fmoc-L-Cys(Trt)-OH; Fmoc-L-Phe-OH; Fmoc-L-Trp(Boc)-OH; Fmoc-L-Thr(tBu)-OH; Fmoc-L-His(Trt)-OH, CH_3_CN, TFA, EtOAc, DMSO, DIC, Oxyma Pure, DMF, DMSO/propyl acetate, DMSO/butyl acetate, piperidine, acetic anhydride, DCM, and Et_2_O were purchased from Sigma-Aldrich (Milan, Italy).

### 3.2. Peptide Sequences

LNL: Ac-PKKHPSFWKTSPKKH-NH_2_

MCL2: Ac-PKKHPc(CFWKTC)PKKS-NH_2_

MCL3: Ac-PKKSPc(CFWKTC)PKKH-NH_2_

The peptides LNL, MCL2, and MCL3 were synthesized using fully automated induction-assisted solid-phase peptide synthesis (Gyros Protein Technologies, PurePrep^®^ Chorus, Tucson, AZ, USA). The Fmoc/tBu induction-assisted optimized SPPS protocol [29], performed on a 50 μmol scale, consisted of the following steps:

1. Swelling in EtOAc/DMSO (4:1 *v*/*v*, 3 mL) for 30 min at room temperature.
2. Fmoc removal for 1 min at 90 °C using 20% piperidine in DMF (2 mL).
3. Washings with EtOAc/DMSO (4:1 *v*/*v*, 3 × 2 mL).
4. Couplings: Fmoc-L-aa-OH, DIC, Oxyma Pure (5:5:5), 2 min at 90 °C in sustainable, eco-friendly, green binary solvent mixtures, such as DMSO/propyl acetate or DMSO/butyl acetate, vs. DMF.
5. Washings with EtOAc/DMSO (4:1 *v*/*v*, 2 mL).

Peptide elongation involved repeating the following general protocol for each amino acid adequately orthogonally protected, including Fmoc-L-His(Trt)-OH, Fmoc-L-Lys(Boc)-OH, Fmoc-L-Pro-OH, Fmoc-L-Cys(Trt)-OH, Fmoc-L-Thr(tBu)-OH, Fmoc-L-Trp(Boc)-OH, Fmoc-L-Phe-OH, and Fmoc-L-Ser(tBu)-OH.

Both deprotection and coupling reactions occurred in a glass vessel under mechanical mixing and nitrogen bubbling. Following the last cycle of Fmoc removal, the N-terminal position was acetylated using a 10% *v*/*v* solution of acetic anhydride in DMF (2 mL).

After washing with DMF and DCM or with isopropyl alcohol (3 × 3 mL/g resin), the resin was dried under *vacuum*. Peptide cleavage from the resin, along with deprotection of acid-labile amino acid side chains, was achieved by treating the peptide-resin with a cocktail of TFA/TIS/DODT/H_2_O (10 mL, 94:2.5:2.5:1) for 2 h at room temperature under mechanical shaking. The resin was then filtered and rinsed with fresh TFA. The cleavage mixture was precipitated by adding ice-cold Et_2_O (35 mL). The resulting crude peptide precursor was washed with ice-cold Et_2_O (35 mL) and dried under *vacuum*.

The crude peptides with free cysteine residues MCL2 and MCL3 were solubilized in a mixture of water and CH_3_CN (1:1) and stirred for approximately 15 min to facilitate the formation of intramolecular disulfide bonds. After complete dissolution, additional water was added to the reaction mixture to achieve a final concentration of 5.3 mM. Initially, the pH that was measured at 2.5 was adjusted to 9.5 by adding NH_4_OH (7.5%). H_2_O_2_ (0.8 eq) was then added, and the mixture was mechanically stirred for 2 h at 350 rpm at room temperature. Finally, the reaction was quenched by adding TFA to readjust the pH to 2.5. The reaction mixture was subsequently lyophilized without further evaporation. The syntheses were also planned to use sustainable green solvents, considering that on 12th December 2023, the European Commission took regulatory action to amend Annex XVII of REACH, imposing restrictions on the use of DMF owing to its high toxicity and impact on health [29,30].

The crude peptides were purified via reverse-phase flash chromatography, monitored by a UV detector (Teledyne ISCO CombiFlash NextGen 300+, Lincoln, NE, USA) using a SNAP Ultra C18 column (30 g), with a column volume (CV) of 45 mL; flow rate set at 25 mL/min; eluents composed of 0.1% TFA in H_2_O (A) and 0.1% TFA in CH_3_CN (B); gradient: 3 CV A, followed by 10 CV from 0% to 50% B, and ending with 3 CV of B.

Subsequent lyophilization yielded the desired peptides as a white powder, with HPLC purity of 98.5% for LNL, 98.5% for MCL2, and 99.1% for MCL3.

The eluted fractions were subjected to analysis by RP-UHPLC-MS using a Thermo Scientific Ultimate 3000 (Thermo Fisher Scientific, Waltham, MA, USA) equipped with a diode array detector and a Thermo Scientific MSQ PLUS (Thermo Fisher Scientific, Waltham, MA, USA) and employing a C18 Waters Acquity CSH™ column (130 Å, 1.7 µm, 2.1 × 100 mm). Operating conditions were maintained at a temperature of 318.15 °K, with a flow rate of 0.5 mL/min, eluents consisting of 0.1% TFA in H_2_O (A) and 0.1% TFA in CH_3_CN (B), and monitoring at λ 215 nm. The gradient ranged from 25% to 60% B in A over 5 min.

LNL: R_t_: 3.22 min, [M + 2H]^2+^ calcd. 938.12; [M + 2H]^2+^ found 938.42. 

MCL2: R_t_: 3.57 min, [M + 2H]^2+^ calcd. 928.14; [M + 2H]^2+^ found 928.38. 

MCL3: R_t_: 3.58 min, [M + 2H]^2+^ calcd. 928.14; [M + 2H]^2+^ found 928.35.

RP-UHPLC traces and ESI-MS spectra of LNL, MCL2, and MCL3 are reported in the Supplementary Materials.

### 3.3. Potentiometric Measurements

Potentiometric measurements were performed at 25 °C using a semi-micro combination electrode (Metrohm AG, Herisau, Switzerland) and a pH-meter system calibrated with HCl at a hydrogen ion concentration. The pH-metric titrations were carried out in an ionic strength I = 0.1 mol L^−1^ KCl/HCl solution with a sample volume of 1.5 mL at a ligand concentration of 6 × 10^−4^ mol L^−1^. A 2 mL micrometer syringe was used to introduce KOH. It included 0.1 mol L^−1^ of KOH. The pH range of 2.5 to 11.5 served as the measurement range. The titration curves, HYPERQUAD [31], and SUPERQUAD [32] softwares were used to calculate the stability constants and stoichiometry of the complexes.

### 3.4. Spectroscopic Measurements

Using a Varian Cary 50 Bio spectrophotometer (Varian Inc., Paolo Alto, CA, USA), visible spectra in the 300–800 nm range were captured at 25 °C. For all systems under investigation, magnetic circular dichroism (MCD) spectra were captured at 25 °C using a Jasco J-1500 spectrometer (Jasco Corporation, Ishikawa-machi, Hachioji-shi, Tokyo Japan). The MCD spectra were captured using a Permanent Magnet PM-491 accessory and collected in a magnetic field of +1.6 T in the N/S field direction. All spectroscopic measurements were collected in the pH range of 2.5 to 11. The reactions were carried out in a 0.3 mol L1 KCl solution with a ligand concentration of 6 × 10^−4^ mol L^−1^. 2.0 mL was used for each sample. With the aid of a Mettler Toledo pH meter and modest additions of concentrated KOH and HCl solutions, the pH values were determined. The electron paramagnetic resonance (EPR) spectra were recorded on a Bruker ELEXSYS E500 CW-EPR X-Band spectrometer equipped with an ER 036TM NMR Teslameter and an E 41 FC frequency counter (Bruker AXS GmbH, Karlsruhe, Germany). A microwave power of 10 mW, a modulation frequency of 100 kHz, a modulation amplitude of 10 G, a time constant of 82 ms, and a conversion time of 164 ms were adopted. The samples were sealed in quartz tubes and placed inside a standard EPR quartz tube for measurements.

## 4. Conclusions

We report herein the study of four analogues of the bicyclic peptide with the sequence c(PKKHP-c(CFWKTC)-PKKH) (BCL) and the role of the structural aspects on the coordination abilities of the bi-cyclopeptide with the unbinding cycle c(CFWKTC). The analysis of the potentiometric results has shown that the bi-cyclic structure promotes the involvement of the side chain imidazole donors in the Cu(II) binding. Based on the results obtained for the mono-cyclic analogues containing two His residues (MCL1: Ac-PKKHPc(CFWKTC)PKKH-NH_2_) and one His residue (MCL2: Ac-PKKHPc(CFWKTC)PKKS-NH_2_ and MCL3: Ac-PKKSPc(CFWKTC)PKKH-NH_2_), respectively, it can be assumed that the coordination of the histidine moiety as an anchoring group is promoted by its location in the peptide sequence further from the nonbinding cycle that strongly influences the involvement of the amide donors in the Cu(II) coordination. The spectroscopic studies, based on MCD and EPR, support the hypothesis that neither the direct interaction of the tryptophan side chain with the Cu(II) ion nor other groups affect the appearance of an unusual band below 500 nm in the UV–vis spectrum.

## Figures and Tables

**Table 1:** The protonation and stability constants of Cu(II)-complexes of BCL [25], MCL1, and LNL peptides.

**Species**	**BCL** [22]		**MCL1**		**LNL**	
	***logβ***	***logK***	***logβ***	***logK***	***logβ***	***logK***
AH	10.78	10.78	10.93 ± 0.10	10.93	10.22 ± 0.15	10.22
AH_2_	21.23	10.45	21.26 ± 0.06	10.33	20.31 ± 0.09	10.09
AH_3_	31.47	10.24	31.42 ± 0.09	10.16	30.49 ± 0.08	10.18
AH_4_	41.33	9.86	41.07 ± 0.05	9.65	39.71 ± 0.04	9.22
AH_5_	50.59	9.26	50.07 ± 0.06	9.00	49.02 ± 0.05	9.31
AH_6_	57.11	6.52	56.74 ± 0.07	6.67	55.72 ± 0.05	6.70
AH_7_	62.78	5.67	62.17 ± 0.07	5.43	61.32 ± 0.05	5.60
**Cu(II) complexes**						
	**BCL** [22]		**MCL1**		**LNL**	
	***logβ***	***logK***	***logβ***	***logK***	***logβ***	***logK***
${\mathrm{CuH}}_{6}L$	60.91	4.64	-	-	-	-
${\mathrm{CuH}}_{5}L$	56.27	6.89	54.59 ± 0.02	13.97	53.10 ± 0.06	6.09
${\mathrm{CuH}}_{4}L$	49.38	8.03	-	-	47.01 ± 0.05	65.6
${\mathrm{CuH}}_{3}L$	41.35	-	40.62 ± 0.06	18.47	-	-
CuHL	-	-	22.15 ± 0.09	20.32	-	-
${\mathrm{CuH}}_{-1}L$	-	-	1.83 ± 0.09	21.31	-	-
${\mathrm{CuH}}_{-3}L$	-	-	−19.48 ± 0.05	-	−18.59 ± 0.06	-
${\mathrm{Cu}}_{2}H_{4}L$	52.59	13.06	-	-	-	-
${\mathrm{Cu}}_{2}H_{2}L$	39.53	7.29	38.48 ± 0.03	7.80	38.03 ± 0.04	7.54
${\mathrm{Cu}}_{2}\mathrm{HL}$	32.14	9.35	30.68 ± 0.04	8.61	30.49 ± 0.05	8.72
${\mathrm{Cu}}_{2}L$	22.79	9.09	22.07 ± 0.04	9.70	21.77 ± 0.07	8.77
${\mathrm{Cu}}_{2}H_{-1}L$	13.70	20.27	12.37 ± 0.07	9.52	13.00 ± 0.05	19.08
${\mathrm{Cu}}_{2}H_{-2}L$	-	-	2.85 ± 0.04	20.81	-	-
${\mathrm{Cu}}_{2}H_{-3}L$	−6.57	21.76	-	-	−6.08 ± 0.05	31.26
${\mathrm{Cu}}_{2}H_{-4}L$	-	-	−17.96 ± 0.04	22.60	-	-
${\mathrm{Cu}}_{2}H_{-5}L$	−28.33	-	-	-	-	-
${\mathrm{Cu}}_{2}H_{-6}L$	-	-	−40.56 ± 0.05	-	−37.34 ± 0.05	-

**Table 2:** The protonation and stability constants of Cu(II)-complexes of MCL2 and MCL3 peptides.

**Species**	**MCL2**		**MCL3**	
	***logβ***	***logK***	***logβ***	***logK***
AH	10.32 ± 0.13	10.32	10.63 ± 0.13	10.63
AH_2_	20.99 ± 0.02	10.67	20.62 ± 0.12	9.99
AH_3_	30.71 ± 0.08	9.72	30.79 ± 0.11	10.17
AH_4_	40.58 ± 0.03	9.87	40.53 ± 0.05	9.74
AH_5_	49.40 ± 0.04	8.82	49.38 ± 0.07	8.85
AH_6_	55.13 ± 0.04	5.73	55.13 ± 0.07	5.75
**Cu(II) complexes**				
	**MCL2**		**MCL3**	
	***logβ***	***logK***	***logβ***	***logK***
${\mathrm{CuH}}_{4}L$	45.85 ± 0.14	6.25	46.69 ± 0.04	7.04
${\mathrm{CuH}}_{3}L$	39.60 ± 0.04	8.76	39.65 ± 0.05	9.39
${\mathrm{CuH}}_{2}L$	30.84 ± 0.07	9.30	30.26 ± 0.12	8.82
CuHL	21.54 ± 0.06	20.20	21.44 ± 0.06	19.84
${\mathrm{CuH}}_{-1}L$	1.34 ± 0.06	22.08	1.60 ± 0.07	21.36
${\mathrm{CuH}}_{-3}L$	−20.74 ± 0.08		−19.76 ± 0.08	

## Data Availability

Data are contained within the article and Supplementary Materials.

## Supplementary Materials

The following supporting information can be downloaded at: https://www.mdpi.com/article/10.3390/molecules29102197/s1. Figure S1: RP-UHPLC trace of LNL; Figure S2: ESI-MS spectrum of LNL; Figure S3: RP-UHPLC trace of MCL2; Figure S4: ESI-MS spectrum of MCL2; Figure S5: RP-UHPLC trace of MCL3; Figure S6: ESI-MS spectrum of MCL3.
