# Supplementary material for: The Role of the Unbinding Cycle on the Coordination Abilities of the Bi-Cyclopeptides toward Cu(II) Ions
Source: Molecules. 2024 May 8;29(10):2197. doi: 10.3390/molecules29102197 (PMC11124368; doi:10.3390/molecules29102197)
Supplement: Supplementary file 1 [file molecules-29-02197-s001.zip › molecules-2993538-supplementary.pdf]

SUPPLEMENTARY MATERIALS

The role of the unbinding cycle on the coordination abilities of the bi-cyclopeptides toward Cu(II) ions

Alicja Lisowska[1], Paulina Świątek[2], Filip Dębicki[3], Agnieszka Lewińska[4], Aleksandra Marciniak[5], Lorenzo Pacini[6,7], Anna Maria Papini[6,7*], Justyna Brasuń[5*].

1 Biomolecule Student Science Club, Faculty of Pharmacy, Wroclaw Medical University, 50-556 Wroclaw, Poland
2 Graduate of Faculty of Pharmacy, Wroclaw Medical University, 50-556 Wroclaw, Poland
3 Faculty of Medicine, Medical University of Lodz, 90-647 Lodz, Poland
4 Faculty of Chemistry, Wroclaw University, 50-137 Wroclaw, Poland
5 Department of the Basic Chemical Sciences, Wroclaw Medical University, 50-556 Wroclaw, Poland
6 Interdepartmental Research Unit of Peptide and Protein Chemistry and Biology, University of Florence, 50019 Sesto Fiorentino (FI), Italy
7 Department of Chemistry "Ugo Schiff", University of Florence, 50019 Sesto Fiorentino (FI), Italy

* Correspondence: AMP: annamaria.papini@unifi.it and JB: justyna.brasun@umw.edu.pl

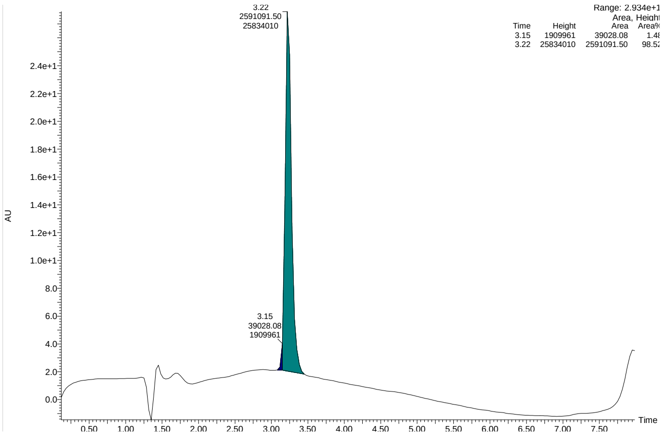

Figure S1: RP-UHPLC trace of **LNL** registered on Thermo Scientific Ultimate 3000 with a diode array detector coupled with a Thermo Scientific MSQ PLUS ESI mass spectrometer. R_t 3.22: LNL. RP-UHPLC-ESI-MS: C18 column Waters Acquity CSH™ (130 Å, 1.7 µm, 2.1 × 100 mm); temperature 45 °C; flow: 0.5 mL/min; eluent: 0.1% (v/v) TFA in H_2O (A) and 0.1% (v/v) TFA in CH_3CN (B), λ 215 nm, gradient: 25−60% B in 5 min.

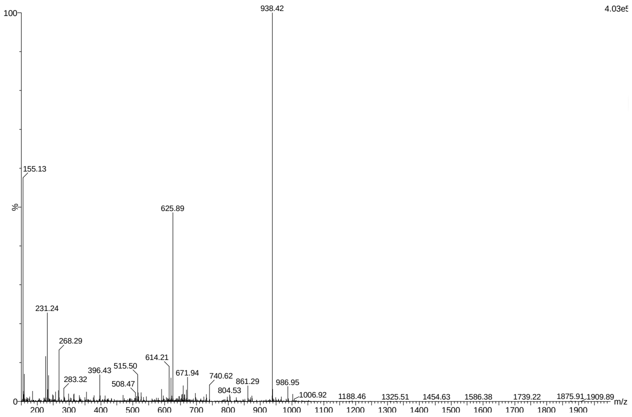

Figure S2: ESI-MS spectrum of **LNL** registered on a Thermo Scientific MSQ PLUS ESI mass spectrometer. ESI-MS (m/z): $[M+2H]^{2+}$ calcd. 938.12; $[M+2H]^{2+}$ found 938.42.

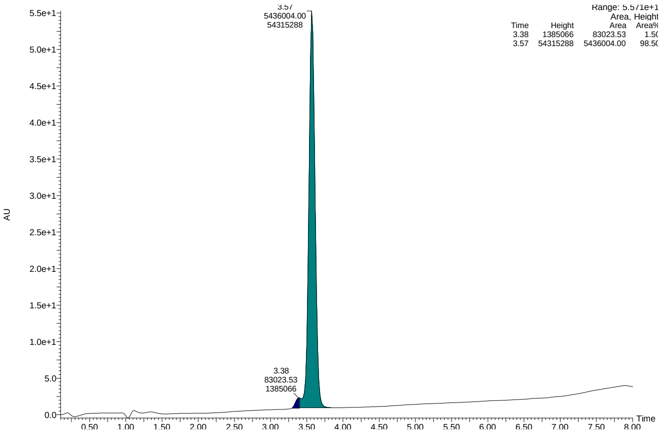

Figure S3: RP-UHPLC trace of **MCL2** registered on Thermo Scientific Ultimate 3000 with a diode array detector coupled with a Thermo Scientific MSQ PLUS ESI mass spectrometer. R_t 3.57: MCL2. RP-UHPLC-ESI-MS: C18 column Waters Acquity CSH™ (130 Å, 1.7 µm, 2.1 × 100 mm); temperature 45 °C; flow: 0.5 mL/min; eluent: 0.1% (v/v) TFA in H_2O (A) and 0.1% (v/v) TFA in CH_3CN (B), λ 215 nm, gradient: 25−60% B in 5 min.

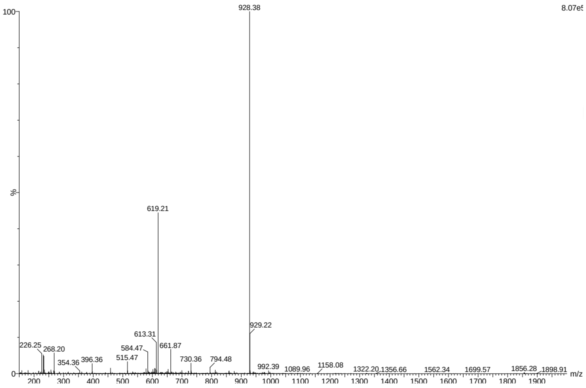

Figure S4: ESI-MS spectrum of **MCL2** registered on a Thermo Scientific MSQ PLUS ESI mass spectrometer. ESI-MS (m/z): $[M+2H]^{2+}$ calcd. 928.14; $[M+2H]^{2+}$ found 928.38.

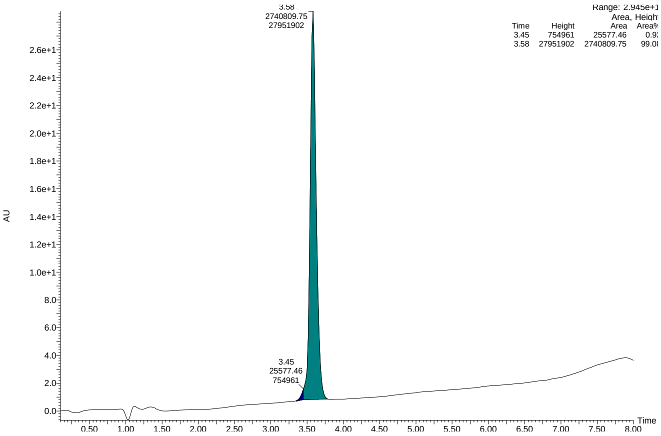

Figure S5: RP-UHPLC trace of **MCL3** registered on Thermo Scientific Ultimate 3000 with a diode array detector coupled with a Thermo Scientific MSQ PLUS ESI mass spectrometer. R_t 3.58: MCL3. RP-UHPLC-ESI-MS: C18 column Waters Acquity CSH™ (130 Å, 1.7 µm, 2.1 × 100 mm); temperature 45 °C; flow: 0.5 mL/min; eluent: 0.1% (v/v) TFA in H_2O (A) and 0.1% (v/v) TFA in CH_3CN (B), λ 215 nm, gradient: 25−60% B in 5min.

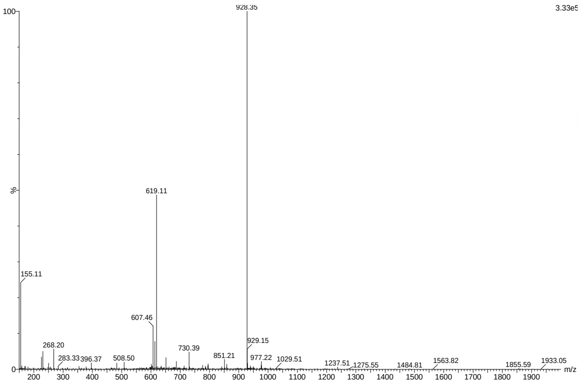

Figure S6: ESI-MS spectrum of **MCL3** registered on a Thermo Scientific MSQ PLUS ESI mass spectrometer. ESI-MS (m/z): $[M+2H]^{2+}$ calcd. 928.14; $[M+2H]^{2+}$ found 928.35.
